# Death of Dr. Thomas W. Blatchford, of Troy, N. Y.

**Published:** 1866-01

**Authors:** 


### Death of Dr. Thomas W. Blatchford, of Troy, N. Y.

We copy the following mention of the life and character of Dr. Blatchford from a Troy paper, as we have no other data upon which to rely, though we have long known Dr. Blatchford as one of the ablest and best physicians:

"We are called upon to announce the death of one of our oldest and most respectable citizens—one of our purest and best men—and one widely known and esteemed, not only in this community, but throughout the State, and even known in the ranks of his profession in Europe as well as in America. THOMAS W. BLATCHFORD, M. D., died at his residence in this city at twenty minutes to one o'clock yesterday, (7th inst.) Although in failing health for some time past, it was only within the last week or two that serious apprehensions were entertained that he would not recover. He was born in England in 1794, and was therefore 71 years old. He came to Troy in 1828, and has been a practicing physician here for about forty years, being at the head of his profession. He was justly esteemed as one of the most eminent physicians in the State. He has during his long professional career contributed numerous monographs to the standard medical publications of the country. One on Hydrophobia, published some years since, attracted general attention in this country, and was translated into several continental European languages and published extensively abroad. He had a thorough medical education, and was an extensive reader in the line of his profession. After a thorough course of schorarly training in this country, he attended the lectures of the famous Sir Astley Cooper in London. It is a singular fact that Doctor Blatchford was a companion of Dr. John W. Webster, who murdered Dr. Parkman in Boston some years since, both in England and America. He was quite young when he came to this country, and resided with his parents at Bridgeport, Conn., until the year 1800, when the family went to Lansingburgh to reside. His father was the Rev. Dr. Samuel Blatchford, and he was settled in Lansingburgh as a Presbyterian clergyman, where he died in 1828. We believe Dr. Blatchford, in addition to his studies under Sir Astley Cooper was also a graduate of the University at Edinburgh. He had held prominent positions in the National and Medical Societies, as well as in other local and general medical associations. Aside from the medical services rendered our citizens, Troy owes him a lasting debt of gratitude for his noble exertions in the cause of education. Prior to 1849 he had been a School Trustee and a School Commissioner, and was prominently identified with the educational interests of the city. He was one of the earnest advocates and fathers of our present excellent system of free schools which was adopted in 1849. He was a member of the first Board of Education in that year and continued an active member until 1856, when the cares of his profession and advancing years induced his resignation. He was one of the earliest Presidents of the

Board, and we believe held that office at the time of his resignation. The Blatchford school in the fourth ward, bears his name as a tribute to his noble exertions in the cause of education. He manifested great interest at all times in the religious and moral questions of the day—was an earnest advocate of temperance—and for a number of years an elder in Dr. Kennedy's church, at which he was a regular attendant. He was a brother of Hon. Richard M. Blatchford, ex-Minister to Rome, and a well-known citizen of New York. He leaves a wife, two sons and a daughter, besides numerous relatives and a large circle of friends and acquaintances to mourn his loss. He had a wide range of practice, and numerous families who were wont to call in his services in the hour of sickness and affliction will greatly miss his consoling and healing visits. Possessed of the noblest qualities, of head and heart, with talents of the highest order, a mind inspired by the noblest impulses, his death will be mourned as a public loss, no less than an irreparable private grief to the immediate circle of relatives and friends who best knew his manly and noble characteristics."

